# Progress in Fevers

**Published:** 1902-10-11

**Authors:** 


### Progress in Fevers.

**Tetanus.**—Recently groups of cases of tetanus following vaccination have occurred in Camden, New Jersey, and in Philadelphia, and the subject of post vaccinal tetanus has therefore been carefully studied by R. N. Willson.[71] He has collected and analysed 52 cases occurring between the years 1839 and 1901. Dr. Willson thinks that, owing to the abnormal demand for lymph created by the recent small-pox epidemic in the United States, some of that supplied was at any rate not potent, yet repeated investigations and inoculation experiments carried out by numerous observers failed to find tetanus bacilli or their spores in any of the suspected lymphs. The care of the vaccination wound is regarded as of very special importance. In nearly every case among the 52 cases collected a large deep ulcer was said to be present, and in all with regard to which information could be collected, some gross neglect of the wound had occurred. Several patients lived in more or less intimate connection with horses or stables. The 52 cases could be grouped to show a time and still more a place incidence, and in this connection Bauer is quoted who records Holland's report that while Iceland is almost free from tetanus, the children on the neighbouring island of Heimaey are almost decimated by the disease. Infection in these post-vaccinal cases was almost invariably about the twentieth day after vaccination, that is just at the time when an open wound liable to infection would most often arise from loss of or injury to the scab. Of the 52 cases 41 proved fatal. This severity of type points to a secondary infection of the wound, and not to a primary infection from the lymph, as in acute cases the incubation period is very short. In this country[72] tetanus has followed vaccination perhaps once in 5,000,000 cases, and even in tetanogenic countries the incidence is probably not greater than one in a million cases. The bacilli must be present in the air, for they have been found in abundance in the dust of rooms with wooden floors and in cobwebs used for arresting the bleeding of wounds. Tetanus occasionally supervenes on extensive burns and in these cases infection must necessarily have arisen from the access of infected air or dust or dressings. Infection, if present in the skin, cannot have survived the heat causing the wound. In all cases of tetanus of obscure origin careful examination of the dust present in the houses concerned should be made.

**Influenza.**—T. B. Barringer[73] reports a case of pre-senile or angiosclerotic gangrene precipitated by an attack of influenza. The patient, a married woman of 38, anæmic and suffering from uterine fibroids, and entirely free from specific history, complained for a month or two of neuralgic pains, and later of frequent burning pain in the finger tips of the right hand. Within about a week of contracting a moderately severe attack of influenza the pain grew very severe, redness and swelling supervened, and finally gangrene necessitated amputation of the distal phalanges of the fourth and fifth fingers. A return of all the symptoms three months later necessitated amputation of the third and fourth metacarpal bones. Large continuous doses of potassium iodide with small doses of mercury and the occasional application of bandages soaked in alcohol of 95 per cent. strength, as recommended by Buchner, have since secured freedom from pain and other local symptoms. The patient presented no signs of general arterial disease, the radial artery was quite soft and pulsation well marked in it and in the ulnar artery. Microscopical examination of the vessels and nerves of the amputated parts revealed an extensive endarteritis, a chronic interstitial neuritis, and a round celled infiltration of the tissues and especially of the sheaths of the blood vessels. Many of these infiltration areas were partially necrosed creating practically a condition of miliary abscess. The condition closely resembled that seen in syphilis. The attack of influenza acted by encouraging thrombosis and so leading to complete occlusion of the previously damaged arteries. Eichhorst has collected 19 cases of gangrene following influenza and many cases following other infectious diseases. S. Mackenzie [74] in considering the personal factor in diseases of microbic origin protests against the tendency in medicine to-day to attach too much importance to the microbic germ or seed and too little importance to the body, the soil in which the germ takes root. For the correct understanding and treatment of disease of microbic origin the careful study of the soil is as essential as that of the seed. He considers the factor of natural resistance as shown in various diseases. In influenza the natural resistance or personal factor is so small as to be practically *nil* as far as susceptibility goes; neither age, sex, class nor race affords exemption from attack when the disease is pandemic. But the effects of the disease differ widely in different individuals, and although more generally severe in the aged and weakly and in those suffering from cardiac or pulmonary disease, the complications are many and various and include abscess of the brain, purulent otitis, phlebitis, affections of the heart and nervous system, etc. In other diseases, as in rheumatic fever and appendicitis account must be taken of this personal factor, but pre-eminently is this the case in regard to tuberculosis, the bacillus of which is so widely disseminated that no one can be exempt from the risk of infection.

**Continued Fever due to the bacillus enteritidis of Gaertner.** A case is reported by Craig and White.[75] The patient, a temperate man of 22 years, suffered from fever, diarrhœa, the stools being thin and yellowish, enlarged spleen, a copious eruption of rose spots, and violent delirium. Widal's reaction was obtained in a dilution of 1 in 25. Clinically the case was regarded as typhoid, or at one time as being typhus fever. The necropsy revealed an enlarged spleen and inflammation of the mucous membrane throughout the small intestine, but no affection of Peyer's patches. From the spleen Professor White obtained in pure culture an organism resembling the typhoid bacillus, but considered by him to belong to the B. enteritidis group. The organism was a motile bacillus, decolourised by Gram's method, it did not coagulate milk, or liquefy gelatine, it fermented glucose, but not glycerine. Serious illness has followed the eating of meat containing these organisms. The title "Gaertner group" has been given by Durham to the intermediate bacilli of the typhoid-colon group.[76] This group includes B. enteritidis, which produces epidemics of meat poisoning; B. psittacosis, which gives rise to a peculiar epidemic, broncho-pneumonia with typhoid symptoms; B. paratyphosus, which produces a disease closely resembling typhoid fever; B. choleræ suis and others. N. E. Brill claims to have pointed out some years ago the existence of a disease clinically resembling typhoid fever, but not giving Widal's reaction. The bacillus isolated in 14 similar cases reported by other observers, although resembling B. typhosus in many biological features, produced gas in glucose media.

71 Brit. Med. Jour., May 3. 72 Lancet, March 1, p. 607. 73 Med. Rec.. March 1. 74 Lancet, May 31. 75 Brit. Med. Jour., May 24, p. 1270 76 Med. Rec., March 29, p. 515.

